# Supplementary material for: Epithelial-Mesenchymal Transition is Associated with Altered Immune Composition and Cytotoxic Function in Triple-Negative Breast Cancer
Source: bioRxiv. 2025 Oct 23:2025.10.22.683714. Preprint. [Version 1] doi: 10.1101/2025.10.22.683714 (PMC12633317; doi:10.1101/2025.10.22.683714)
Supplement: Supplement 4 [file media-4.pdf]

Supplementary Table 1. EMT-related genes

ZEB1	TNFRSF21	EVPL	KRT19	KDF1	TMEM30B
LIX1L	TMEM45B	FXYD3	GRHL1	CDS1	CLDN7
VIM	MPP7	CLDN4	BSPRY	CHEK2	EPCAM
AXL	CHAF1B	CRB3	C1orf116	MPZL2	SCNN1A
MMP2	SSH3	TSKU	S100A14	PATJ	CDH1
ANTXR2	TC2N	MAPK13	S100A11P1	ESRP1	FZR1
XXYLT1	MUC1	GALNT3	SPINT2	TMC4	
FN1	EPPK1	STAP2	ANKRD22	ITGB6	
NRP1	SHROOM3	DSP	ST14	TMEM125	
TGFBI	EPN3	ELMO3	GRHL2	EPHA1	
GALNT5	PRSS22	KRTCAP3	PRR5	ENPP5	
PPARG	AP1M2	MAL2	TJP3	EPB41L5	
HNMT	SH3YL1	F11R	TACSTD2	ERBB3	
CARD6	KLC3	ADGRF1	CDH3	RAB25	
RBPMS	SERINC2	ADGRG1	CDH15	PRSS8	
